# The effect of oat bran consumption on gestational diabetes: a randomized controlled clinical trial

**DOI:** 10.1186/s12902-021-00731-8

**Published:** 2021-04-13

**Authors:** Zahra Barati, Mina Iravani, Majid Karandish, Mohammad Hosein Haghighizadeh, Sara Masihi

**Affiliations:** 1 School of Nursing and Midwifery, Ahvaz Jundishapur University of Medical Sciences, Ahvaz, Iran; 2 Reproductive Health Promotion Research Center, Midwifery Department, School of Nursing and Midwifery, Ahvaz Jundishapur University of Medical Sciences, Ahvaz, Iran; 3 Department of Nutritional Sciences, School of Paramedical Sciences, Nutrition and Metabolic Disease Research Center, Ahvaz Jundishapur University of Medical sciences, Ahvaz, Iran; 4 Biostatistics Department, School of public Health, Ahvaz Jundishapur University of Medical Sciences, Ahvaz, Iran; 5 Department of Obstetrics and Gynecology, School of Medicine, Fertility Infertility and Perinatology Research Center, Imam Khomeini Hospital, Ahvaz, Iran

**Keywords:** Oat bran, Fasting blood sugar, Two-hour post-prandial (2hpp) glucose

## Abstract

**Background:**

Gestational diabetes is the most common medical complication in pregnancy, and it has many side effects for the mother and the fetus. The aim of this study was to evaluate the effect of oat bran consumption on gestational diabetes.

**Methods:**

This study is a randomized clinical trial that was performed on 112 women with gestational diabetes treated with diet. Participants were randomly divided into two groups of 56. Participants in both groups were given a diet for gestational diabetes. In addition to the diet, the intervention group received 30 g of oat bran daily for 4 weeks at lunch and dinner. Tests of fasting blood glucose and two-hour postprandial (2hpp) glucose were taken from both groups: before the intervention, and 2 and 4 weeks after the start of the intervention. Data analysis was performed using SPSS statistical software (version 22) using independent t-test, as well as Chi-square and Mann-Whitney tests. *P* values less than 0.05 were considered statistically significant.

**Results:**

There was no statistically significant difference between the two groups in terms of mean blood glucose before the intervention, while 2 and 4 weeks after the intervention, mean fasting blood glucose and two-hour postprandial (2hpp) glucose decreased significantly in the intervention group compared with the control group (*P* < 0.001).

**Conclusion:**

Based on the results of this study, the addition of oat bran to the standard diet for pregnant women with gestational diabetes reduced fasting blood glucose and two-hour postprandial (2hpp) glucose. More detailed studies with higher sample sizes are recommended to prove the effectiveness of this valuable dietary supplement.

**Trial registration:**

**IRCT registration number:** IRCT20191220045828N1.

**Registration date:** 2020-04-18. Registered while recruiting.

## Background

Diabetes is a huge problem for people’s health. This metabolic disease is associated with chronic hyperglycemia, impaired insulin secretion or function, and impaired protein and fat metabolism [1]. One type of diabetes is gestational diabetes that is characterized with glucose intolerance with variable severity which first begins or is diagnosed during pregnancy [2]. Approximately, 3–5% of pregnancies are complicated by gestational diabetes [3]. By 2030, diabetes will be the seventh leading cause of death in the world [4]. Some women with gestational diabetes actually have overt diabetes that has not been diagnosed [5]. The prevalence of gestational diabetes is affected by different diagnostic criteria and varies in different populations from about 3.6% in Northern Europe to 6.3% in Italy [6]. The lowest prevalence (1%) is in Tanzania and Singapore. South India has been reported to be 0.6% and in the UK and Northern Europe, the figure is 5.2% [7].

Overall, 4.7% of pregnant women in Iran suffer from this condition [8]. The prevalence of gestational diabetes and impaired glucose tolerance in Ahvaz in 2012, according to the Diabetes Research Center of Ahvaz Jundishapur University of Medical Sciences, was 7.4 and 4.3%, respectively [9].

Gestational diabetes is one of the most common causes of delivery-related complications for both the mother and the fetus [10]. Studies have shown that gestational diabetes increases the risk of developing polyhydramnios, infections (candidal vulvovaginitis, urinary tract and respiratory infections, and puerperal sepsis), gestational hypertension, pyelonephritis, increased risk of developing type 2 diabetes in the future, diabetic ketoacidosis, and increased rate of cesarean section [11, 12].

Fetal and neonatal complications include: unexplained fetal death, low birth weight, fetal macrosomia, maternal and neonatal birth trauma, stillbirth, shoulder dystocia, neonatal hypoglycemia, fetal growth retardation, preterm delivery, respiratory distress syndrome, neonatal immaturity, neonatal hypocalcemia, and risk of future diabetes [11–13].

Available treatments for diabetes include bringing changes in lifestyle, relying on exercise, nutrition, oral medications, and insulin [14].

Prior to the discovery of insulin and conventional drugs, diabetics were treated with herbs and traditional medicines. During the last 10 to 20 years, many clinical and laboratory studies have been conducted on medicinal plants, some of which have shown significant effects in lowering blood sugar [15]. In this regard, the FDA estimates that there are currently more than 29,000 herbal remedies, vitamins or dietary supplements, and more than 1000 are being added every month [16].

*Avena sativa* L., commonly known as Oats, belongs to the Gramineae family, which is an herbaceous plant with a stem of 50 to 70 cm and even one meter high that has barley-like florets inside two pointed glumes [17].

Beta-glucan is a soluble fiber available from barley and oat grains, which has been reported to play a beneficial role in reducing insulin resistance, dyslipidemia, hypertension, and obesity [18].

The physiological effect of dietary fiber depends on its ability to ferment in the large intestine, and this is influenced by the properties of the fiber, including its solubility [19, 20].

Cellulose and beta-glucans are non-starchy polysaccharides of oats and are important dietary fibers in cereals. Most oat fibers are in its hull [21].

In a study by Tapola et al. (2005) which aimed to investigate the glycemic response of oat bran products in type 2 diabetic patients, the results showed that oat bran can act as an effective substance to reduce the glycemic response in type 2 diabetics [22].

Rezvani et al. (2011) carried out a double-blind study that aimed to compare the effect of consumption of real oatmeal bread with conventional barley bread offered in Tehran on serum glucose and fat levels in patients with dyslipidemia. The study was conducted on 40 people with type 2 diabetes, and results showed a significant reduction in fasting cholesterol and blood sugar [23].

However, Geoch et al. (2008) found no significant difference between glycemic control and insulin response in oatmeal consumption compared to the standard diet [24].

The prevalence of gestational diabetes and its complications for the mother, the fetus and the neonate is increasing. Also, some drugs used for the treatment of this disorder are not without side effects, and some pregnant mothers refuse to use insulin. In spite of all of these and given the positive impact of oats on blood sugar in non-pregnant people, no study has thus far been done on the effect of oat bran on gestational diabetes. Therefore, the present study was conducted to investigate the effect of using oatmeal on gestational diabetes.

## Methods

The present study is a randomized clinical trial that was conducted to investigate the effect of oat bran consumption on gestational diabetes.

Sampling started on 04/02/2020 ended on 04/06/2020. This study was approved by the Ethics Committee of Ahvaz Jundishapur University of Medical Sciences (approval code: IR.AJUMS.RE 784). This study was also registered in the Iranian Clinical Trial Registration Center under the number IRCT20191220045828N1. Participants were selected from among pregnant women referring to the Health Center of East Ahvaz and the Midwifery Clinic of Imam Khomeini Hospital in Ahvaz, Iran.

Inclusion criteria: having a cell phone, age 18 to 35 years, having impaired blood sugar (fasting blood sugar equal to or greater than 92, one hour GCT equal to or greater than 180, or blood sugar 2 h after consuming 75 g of glucose equal to or more than 153 mg / dL), being screened in 24–28 weeks of gestation.

Exclusion criteria include: a history of overt diabetes or a disease that interferes with the research process such as: diseases of liver or kidney, mental illness, stroke, allergy to oatmeal, history of stillbirth, gestational diabetes, macrosome births, and a family history of diabetes.

Sampling was performed by referring to the selected centers and briefing the related authorities about the research. After identifying the eligible women from among the pregnant women referring to the health centers using the permuted block randomization method (blocks of four), informed consent was obtained from the participants. The sample size was determined based on the sample size estimation using the two mean comparison formula.

According to the test power of 90% and confidence level of 95%, 51 women were assigned to each group. Considering a 10% attrition rate, 56 women were considered for each group. Eligible individuals were randomly divided into two groups of case and control, 56 each, using the permuted block randomization method (blocks of four) in a ratio of 1: 1. In order to increase the reliability in preventing selection bias, the allocation concealment method was used, and the sample codes were placed in closed envelopes.

Data collection tools in this study included: demographic information form and a 24-h dietary recall (for 72 h) which were provided to participants and completed by them. (The latter was prepared by studying books and authoritative scientific sources, and to evaluate its validity, the content validity method was used. For this purpose, the form was provided to 10 professors of the School of Nursing and Midwifery of Ahvaz Jundishapur University of Medical Sciences. The form was modified based on their recommendation).

Before the intervention, blood samples were taken from all women to determine fasting blood sugar and blood sugar one and two hours after administration of 75 g of oral glucose. Then, the participants were randomly divided into two groups of 56 (Cases: standard diet with oat bran; Controls: standard diet without consumption of oat bran) according to the inclusion criteria. Both groups received the diet for 4 weeks. In this study, 56 people in the intervention group, in addition to receiving the standard diet for gestational diabetes, were given 600 g of oat bran (3 packs of 200 g, OAB™ by Golden Light Cup Company). Diabetic women in the experimental group consumed 30 g (equivalent to 3 to 4 tablespoons half full) of oat bran with lunch and dinner daily for four weeks. In order to follow up the pregnant mothers, a phone call was made every other night as a reminder of consumption, and phone calls were made to inquire about any allergies to oatmeal.

Fasting blood glucose (*FBS)* and two hours after breakfast *(2hpp)* glucose were *checked 2 and 4 weeks after the start* of the intervention in both groups of pregnant mothers (3 cc of venous blood samples were taken from the subjects after 8–12 h of fasting by referring to the laboratory).

Independent t-test, repeated measure test and Mann-Whitney test were used to compare the two groups. At the end of the study, the data were analyzed using SPSS version 22. *P* values less than 0.05 were considered statistically significant.

## Results

**Fig. 1:**
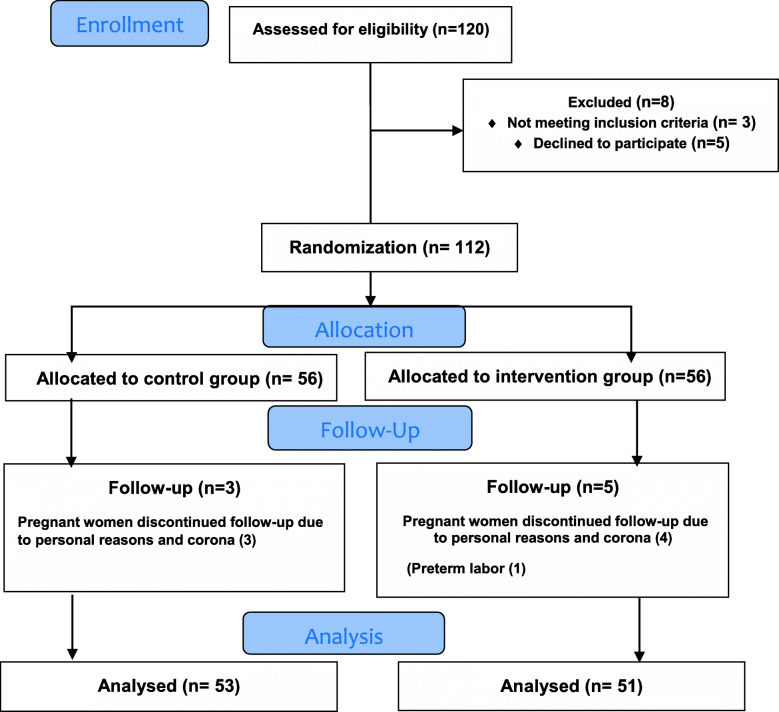
Flowchart of the progress through the phases of the trial

Five people in the oat bran group and 3 people in the control group were excluded from the study due to unwillingness to cooperate in the study. Therefore, the results were analyzed with 51 subjects in the oat bran group and 53 subjects in the control group (Fig. 1).

Based on the results of this study, the two groups were similar and did not have a statistically significant difference in terms of mean age (*p* = 0.507), body mass index (*p* = 0.273), ethnicity (*p* = 0.058), education (0.115), occupation (*p* = 0.007), place of residence (*p* = .990), and type of home ownership (*p* = 0.061) (Table 1).

**Table 1:** Descriptive data of demographic variables

Variable		Control group (n=53)	Intervention group (n=51)	***P***-value^**a**^
		Mean ± SD	Mean ± SD	
Mother’s age (year)		28.72 ±4.13	29.23± 3.80	0.507
BMI(kg/*m*^2^)		23.04± 1.29	22.75 ± 1.38	0.273
		**Number (percentage)**	**Number (percentage)**	***P*-value**^**b**^
Ethnicity	Persian	(66.0)35	)82.4(42	0.058
	Arab	(34)18	(17.6)9	
Education	diplom NO	)28.3(15	(13.7)7	0.11
	Diploma	(34.0)18	)31.4(16	
	University degree	)37.7)20	(54.9)28	
Occupation	Employed	(88.7)47	(66.7(34	0.007
	Housewife	)11.3(6	(33.3)17	
Place of residence	City	)98.15(52	(100)51	<990
	Village	(1.9)1	0(0)	
Type of house ownership	Owner	)82.7(43	)66.7(34	0.061
	Tenant	(17.3)9	(33.3)17	

a: Based on Mann Whitney test

b: Based on chi-squared test

Also, as far as mean gestational age (*p* = 0.277), parity (*p* = 0.433), gravidity (*p* = 0.208) and number of children (*p* = 0.352) were concerned, no statistically significant difference was observed between the two groups (Table 2).

**Table 2:** Descriptive data of midwifery variables

Variable		Control group (n=53)	Intervention group (n=51)	***P***-value^**a**^
		Mean ± SD	Mean ± SD	
Gestational age (day)		5.11 ± 188.92	6.00 ± 190.11	0.277
		**Number (percentage)**	**Number (percentage)**	***P*-value**^**b**^
Parity	0	(23(43.4	(28(54.9	0.433
	1	(16(30.2	(14(27.5	
	2	(14(26.4	(9(17.6	
Gravidity	1	(20(73.7	(28(54.9	0.208
	2	(35.8)19	(27.5)14	
	3	(26.4)14	(17.6)9	
Number of children	0	(37.7)20	(51.0)26	0.352
	1	(35.8)19	(31.4)16	
	2	(26.4)14	(17.6)9	

a: Based on Mann Whitney test

b: Based on chi-squared test

Before the intervention, based on the results of independent t-test, the two groups were not significantly different in terms of fasting blood sugar (*p* = 0.549), blood sugar one hour after consuming 75 g of oral glucose (*p* = 0.555), and two hours after consumption of 75 g of oral glucose (*p* = 0.188) (Table 3).

**Table 3:** Comparison of mean fasting blood sugar and oral glucose tolerance test before intervention in intervention and control groups

Variable	Intervention group (n = 51)	Control group (n = 53)	***P***-value
Fasting blood sugar (mg/dl)	104.69 ± 7.51	105.68 ± 8.24	0.549^a^
Blood sugar one hour after glucose consumption (mg/dl)	182.10 ± 6.70	180.70 ± 7.81	0.555^a^
Blood sugar two hours after glucose consumption (mg/dl)	156.21 ± 4.40	157.68 ± 6.60	0.188^c^

a: Based on Mann Whitney test

c: Based on t test

According to Table 4, the mean fasting blood glucose before the intervention was (104.69 ± 7.51) in the oat bran group and (105.68 ± 8.24) in the control group, and their difference was not statistically significant.

**Table 4:** Comparison of mean fasting blood sugar before intervention and two and four weeks after intervention in intervention and control groups

Variable	Intervention group (n = 51)	Control group (n = 53)	***P***-value
Fasting blood sugar before intervention (mg/dl)	104/69 ± 7/51	105.68 ± 8.24	0.549^a^
Fasting blood sugar two weeks after intervention (mg/dl)	88.49 ± 2.07	99.07 ± 8.14	> 0/001^c^
Fasting blood sugar four weeks after intervention (mg/dl)	84.59 ± 2.84	92.77 ± 4.97	> 0/001^c^
*P*-value	> 0/001^d^	> 0/001^d^	

a: Based on Mann Whitney test

c: Based on t test

d: Based on analysis of variance with repeated measures

Two weeks after consuming oat bran, the mean fasting blood sugar was (88.49 ± 2.07) in the intervention group and (99.07 ± 8.14) in the control group, which was statistically significant (*P* < 0.001).

Also, Table 4 shows that the mean fasting blood sugar 4 weeks after the start of the intervention was (92.77 ± 4.97) in the control group and (84.59 ± 2.84) in the oat bran group, which was significantly different (*p* < 0.001).

According to Table 5, the mean two-hour postprandial (2hpp) glucose before the intervention was (157.68 ± 6.60) in the control group and (156.21 ± 4.40) in the intervention group. Two weeks after the start of oat bran consumption, the mean the two-hour postprandial (2hpp) glucose was (122.17 ± 3.91) in the control group and (115.37 ± 3.14) in the oat bran group, which was significantly different (*p* < 0.001).

**Table 5:** Comparison of mean fasting blood glucose before intervention and blood glucose 2 h after eating two and four weeks after intervention in intervention and control groups

Variable	Intervention group (n = 51)	Control group (n = 53)	***P***-value
Blood sugar 2 h after eating before intervention (mg/dl)	156.21 ± 4.40	157.68 ± 6.60	0.188^c^
Blood sugar 2 h after eating two weeks after intervention (mg/dl)	115.37 ± 3.14	122.17 ± 3.91	> 0/001^c^
Blood sugar 2 h after eating four weeks after intervention (mg/dl)	104.04 ± 5.48	117.49 ± 11.34	> 0/001^c^
*P*-value	> 0/001^d^	> 0/001^d^	

c: Based on t test

d: Based on analysis of variance with repeated measures

Also, according to Tables 5, 4 weeks after start of the consumption of oat bran, the mean two-hour postprandial (2hpp) glucose was (117.49 ± 11.34) in the control group and (104.04 ± 5.48) in the oat bran group, which was significantly different (*P* < 0.001).

According to Table 6, by comparing the 3-day dietary recall in both groups, the two groups were not significantly different in terms of average fat intake (*p* = 0.67), average carbohydrate intake (*p* = 0.28), protein intake (*p* = 0.23) and fiber intake (*p* = 0.46).

**Table 6:** Comparison of average daily intake of calories, carbohydrates, protein, fat and fiber based on body mass index and maternal weight in intervention and control groups.

Variable	Standard diet (***n*** = 53)	Standard diet plus oatmeal(***n*** = 51)	***P***-value
Average daily calories consumed (kilocalories)	2081.91	2028.28	0.22
Average daily carbohydrates consumed (gram)	241.79	233.99	0.28
Average daily proteins consumed (gram)	84.78	80.83	0.23
Average daily fat consumed (gram)	59.21	58.80	0.67
Average daily fiber consumed (gram)	24.93	29.55	0.04
Average daily oatmeal consumed (gram)	0	30	0.0001

## Discussion

The present study aimed to determine the effect of oat bran consumption on gestational diabetes. According to our results, inclusion of oat bran into diet for 4 weeks compared to conventional diet alone, has a positive effect on lowering fasting blood sugar and two-hour postprandial (2hpp) glucose, 2 and 4 weeks after treatment.

Steinert et al. (2015) examined the effect of oatmeal consumption in pre-meal water on the glycemic response in healthy individuals. They found that oatmeal was effective in lowering blood sugar in individuals [25]. Rezvani et al. (2011) compared the effect of real oatmeal bread and barley bread available in the market on serum glucose and fat levels of patients with type 2 diabetes. According to their results, consumption of 250 g of oatmeal bread for 3 weeks in 36 patients with type 2 diabetes, was effective in lowering fasting blood sugar and cholesterol, which is consistent with the results of the present study [23]. Results of a meta-analysis study by Bio et al. (2014) conducted on 15 articles with 673 participants, showed the beneficial effect of oatmeal on blood sugar. In their study, consumption of oatmeal significantly reduced fasting insulin but there was a slight decrease in fasting glucose [26].

Pick et al. (1996) conducted a 24-week crossover study consisting of two 12-week periods on 8 men with diabetes with a mean age of 45 years. They showed that oat bran concentrate products provide long-term control of diabetes. The researchers also showed that consuming 9 g of oat bran improves glycemic response, insulin and lipidemia [27]. The results of the mentioned studies are consistent with the results of our study.

Shahedi and Fazilati (2006) conducted an experimental study aimed to investigate the effect of consuming oatmeal in bread on reducing blood sugar and cholesterol in diabetic patients in Isfahan University of Technology. The results of the study showed that consuming 150 g of oat bread (25% oats and 75% wheat) in 21 diabetic subjects in the study, on days 10 and 15 of consumption, led to a significant decrease in the mean fasting blood sugar of 21 participants [28].

Another study by Afaghi et al. (2013) showed that consumption of fiber-rich foods (e.g., wheat bran) is associated with a significant reduction in 2 h post prandial blood sugar in both groups after intervention, which is also consistent with the present study and the use of cereal fiber [29].

However, Geoch et al. conducted a cross-sectional clinical trial from July 2008 to December 2010 to investigate the effect of oatmeal diet on glycemic control, plasma lipids and oxidative stress in the UK. The results of their study showed that there was no significant difference between glycemic control and insulin response in those who consumed oatmeal compared to those having only the standard diet, and this is not consistent with the results of our study [24].

Cellulose and beta-glucan are non-starch polysaccharides of oats and are important dietary fibers in cereals [21]. Oatmeal contains beta-glucan, which can control blood sugar in people [30].

Tapola et al. (2005) investigated the glycemic response of oat bran products in type 2 diabetic patients, and their results showed that oat bran flour due to containing beta-glucan can be used as an effective substance to reduce the glycemic response in type 2 diabetics. The results obtained are consistent with the results of the present study with respect to the reduced blood sugar which can be attributed to the effects of beta-glucan [22].

The beta-glucan of barley lowers blood sugar probably by delaying gastric emptying, and it may also reduce food intake by reducing appetite. Beta-glucan may also lower blood sugar levels by activating the signaling pathways [31].

In a study comparing the acute effect of oatmeal on appetite and satiety compared to a light breakfast, Rebelo et al. showed that oatmeal controls appetite and increases satiety, which can be attributed to the physical and chemical properties of oatmeal’s soluble fiber (Beta-glucan) and its viscosity and hydration properties [32].

In a prospective cohort study on the effect of whole grain consumption on the incidence of type 2 diabetes in the United States, Parker et al. (2013) found that there was an inverse relationship between whole grain consumption and a reduced risk of type 2 diabetes. They also reported that a diet high in whole grains and fiber can protect against diabetes through a variety of mechanisms, including energy depletion [33]. Complications of bran have not been reported in any study on pregnant women [29]. The recommended amount of fiber in pregnancy is 25 to 35 g per day. In many countries the consumed fiber is below 14 g/day for pregnant women. Of course, excessive fiber intake during pregnancy is not recommended due to diarrhea and nutrition loss [34].

Chandalia et al. (2000) conducted a cross-sectional study on 13 patients with type 2 diabetes and found that increased high fiber intake (50 g: 25 g soluble fiber and 25 g insoluble fiber) compared to a medium fiber diet (24 g: 8 g of soluble fiber and 16 g of insoluble fiber) improves glycemic control and is effective in reducing hyperinsulinemia and plasma lipid concentration in patients with type 2 diabetes [35, 36].

Since at the time of writing this manuscript, we did not find a similar study on pregnancy with which we could compare the results of this study, we tried to interpret the findings of this study in relation to the results obtained in studies on type 2 diabetes. The results of most published clinical trials have shown the positive effect of oat bran consumption on lowering blood sugar among diabetics. Therefore, by examining the available scientific evidence and matching them with the existing study, we found that consumption of oat bran can reduce fasting blood sugar and blood sugar two hours after eating.

### Strengths

One of the strengths of the present study is that the present study is the first study to examine the therapeutic effects of oat bran on pregnant women diagnosed with gestational diabetes.

### Limitations

One of the limitations of this study was the impossibility of further follow-up due to the limited time of the project.

### Suggestions

Due to the positive effect of oatmeal on lowering blood sugar, it is recommended that a study be performed with a longer duration, larger sample size of pregnant women with gestational diabetes, as well as pregnant women with overt diabetes.

## Conclusion

Given the increasing prevalence of diabetes in the world and its negative effects on the fetus and mother, which impose a heavy financial burden on communities and families, trying to find treatments with fewer complications, high effectiveness and lower cost is necessary. Therefore, according to the results of this study, this oral supplement can be used safely in the treatment of gestational diabetes. Of course, provided that more detailed studies with larger sample sizes and longer duration are conducted and positive results are achieved.

## Data Availability

The datasets generated and/or analyzed during the current research are not publicly available as individual privacy could be compromised but are available from the corresponding author on reasonable request.

## Abbreviations

FBS: Fasting blood glucose
2hpp: two-hour postprandial
GCT: Glucose Challenge Test
